# Adopting an Intersectionality Lens Within an Undergraduate Gerontology Curriculum

**DOI:** 10.1093/geroni/igaa057.1727

**Published:** 2020-12-16

**Authors:** Margaret Manoogian

**Affiliations:** Western Oregon University, Monmouth, Oregon, United States

## Abstract

Students who plan geriatric/gerontology careers typically learn the biopsychosocial domains of aging. Using intersectionality to understand older adults and family experiences (Calasanti & Kiecolt, 2012), however, offers students a deeper understanding of how aging adults may face interconnected oppressions and inequalities based on race/ethnicity, gender, age, sexual orientation, socioeconomic status, health, and other aspects of social location within micro and macro contexts. Through systematic assessment of student learning outcomes, a planned programmatic approach to integrating intersectionality was adopted within an undergraduate gerontology program. This multifaceted approach will be highlighted including new course development, course case studies, community member voices, practicum applied practices, and research activities.

Calasanti, T. & Kiecolt, K. J. (2012) Intersectionality and aging families. In Blieszner, R., & Bedford, V. H. (Eds.), Handbook of families and aging (pp. 263-286). Santa Barbara, CA: Praeger. Part of a symposium sponsored by Age-Friendly University (AFU) Interest Group.

